# Network motif-based identification of transcription factor-target gene relationships by integrating multi-source biological data

**DOI:** 10.1186/1471-2105-9-203

**Published:** 2008-04-21

**Authors:** Yuji Zhang, Jianhua Xuan, Benildo G de los Reyes, Robert Clarke, Habtom W Ressom

**Affiliations:** 1 Lombardi Comprehensive Cancer Center, Georgetown University, 4000 Reservoir Rd, Washington, DC, USA; 2 Department of Electrical and Computer Engineering, Virginia Polytechnic Institute and State University, 4300 Wilson Blvd., Arlington, VA, USA; 3 School of Biology and Ecology, University of Maine, Orono, ME 04469, USA

## Abstract

**Background:**

Integrating data from multiple global assays and curated databases is essential to understand the spatio-temporal interactions within cells. Different experiments measure cellular processes at various widths and depths, while databases contain biological information based on established facts or published data. Integrating these complementary datasets helps infer a mutually consistent transcriptional regulatory network (TRN) with strong similarity to the structure of the underlying genetic regulatory modules. Decomposing the TRN into a small set of recurring regulatory patterns, called network motifs (NM), facilitates the inference. Identifying NMs defined by specific transcription factors (TF) establishes the framework structure of a TRN and allows the inference of TF-target gene relationship. This paper introduces a computational framework for utilizing data from multiple sources to infer TF-target gene relationships on the basis of NMs. The data include time course gene expression profiles, genome-wide location analysis data, binding sequence data, and gene ontology (GO) information.

**Results:**

The proposed computational framework was tested using gene expression data associated with cell cycle progression in yeast. Among 800 cell cycle related genes, 85 were identified as candidate TFs and classified into four previously defined NMs. The NMs for a subset of TFs are obtained from literature. Support vector machine (SVM) classifiers were used to estimate NMs for the remaining TFs. The potential downstream target genes for the TFs were clustered into 34 biologically significant groups. The relationships between TFs and potential target gene clusters were examined by training recurrent neural networks whose topologies mimic the NMs to which the TFs are classified. The identified relationships between TFs and gene clusters were evaluated using the following biological validation and statistical analyses: (1) Gene set enrichment analysis (GSEA) to evaluate the clustering results; (2) Leave-one-out cross-validation (LOOCV) to ensure that the SVM classifiers assign TFs to NM categories with high confidence; (3) Binding site enrichment analysis (BSEA) to determine enrichment of the gene clusters for the cognate binding sites of their predicted TFs; (4) Comparison with previously reported results in the literatures to confirm the inferred regulations.

**Conclusion:**

The major contribution of this study is the development of a computational framework to assist the inference of TRN by integrating heterogeneous data from multiple sources and by decomposing a TRN into NM-based modules. The inference capability of the proposed framework is verified statistically (*e.g*., LOOCV) and biologically (*e.g*., GSEA, BSEA, and literature validation). The proposed framework is useful for inferring small NM-based modules of TF-target gene relationships that can serve as a basis for generating new testable hypotheses.

## Background

Enormous amount of data has been generated by the use of high-throughput analytical methods in biology during the last two decades. However, the inherited properties of these data create significant problems in their analysis and interpretation. Standard statistical approaches are not powerful enough to dissect data with thousands of variables (i.e., semi-global or global gene expression data) and limited sample sizes (i.e., several to hundred samples in one experiment). These properties are typical in microarray and proteomic datasets [1] as well as other high dimensional data where a comparison is made to biological samples that tend to be limited in number, thus suffering from curse of dimensionality [2].

**Figure 1:**
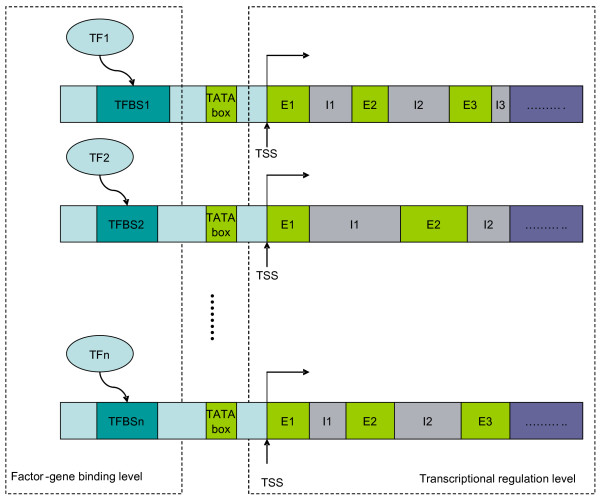
**The gene transcriptional regulatory program**. The gene transcriptional regulatory program can be simplified in two levels. At the factor-gene binding level, the "activated" TFs bind to their specific conserved sequence motifs, called transcription factor binding sites (TFBS). When the binding process is completed, the regulation mechanism instructs the gene transcription from transcriptional start site (TSS) (DNA to mRNA); first part of the central dogma in molecular biology.

One approach to address the curse of dimensionality is to integrate multiple large data sets with prior biological knowledge. This approach offers a solution to tackle the challenging task of inferring transcriptional regulatory networks (TRN). Transcriptional regulation is a process that needs to be understood at multiple levels of description [3,4] (Figure 1) including (1) the factor-target gene interaction, in which transcription factors (TF) activated under certain conditions interact with their conserved binding site sequences; and (2) transcriptional regulation, which explains how the bindings of TFs to their unique recognition sites regulate the expression of specific genes. A single source of information such as gene expression data is aimed at only one level of description (transcriptional regulation level), thus it is limited in its ability to obtain a full understanding of the entire regulatory process. Other types of information such as TF – binding site sequence relationships revealed by genome-wide location analysis [5] provide complementary constraints on the models of regulatory processes. By integrating limited but complementary data sources, we can realize a mutually consistent hypothesis bearing stronger similarity to the underlying causal structures [4]. Among the various types of high-throughput biological data available nowadays, time course gene expression profiles and genomic analysis data are two complementary sets of information that can be used to infer regulatory components. Time course gene expression data are advantageous over typical static expression profiles as time can be used to disambiguate causal interactions. Binding site sequence data based on the analysis of genomic loci, on the other hand, provide high-throughput quantitative information about *in vivo *binding of transcriptional activators to the target regulatory regions of the DNA. Prior biological knowledge generated by geneticists will help guide inference from the above data sets and integration of multiple data sources offers insights into the cellular system at different levels.

A number of researches have explored the integration of multiple data sources (*e.g*., time course expression data and sequence motifs) for TRN inference [6-9]. A typical approach for exploiting two or more data sources uses one type of data to validate the results generated independently from the other (i.e., without data fusion). For example, cluster analysis of gene expression data followed by the identification of consensus sequence motifs in the promoters of genes within each cluster [8]. The underlying assumption behind this approach is that genes co-expressed under varying experimental conditions are likely to be co-regulated by the same TF or sets of TFs. Holmes *et al*. [10] constructed a joint likelihood score based on consensus sequence motif and gene expression data and used this score to perform clustering. Segal *et al*. [11] built relational probabilistic models by incorporating gene expression and functional category information as input variables. Gene expression data and gene ontology (GO) data were combined for TRN discovery in B cell [12]. Computational methodologies that allow systematic integration of data from multiple resources are needed to fully utilize the complementary information available in those resources.

Another way to reduce the complexity of the TRN inference problem is to decompose it into simple units of commonly used network structures. TRN is a network of interactions between TFs and the genes they regulate, governing many of the biological activities in cells. Breaking down the TRN into simplest units of commonly used network architectures helps in understanding complex biological networks. Such patterns of local interconnections are called network motifs (NM) [13]. Since the establishment of the first NM in *Escherichia coli *[14], similar NMs have also been found in eukaryotes including yeast [15], plants, and animals [16-18], suggesting that the general structure of NMs are evolutionarily conserved. One well known family of NMs is the feed-forward loop (FFL) [19], which appears in hundreds of gene systems in *E. coli *[14,20] and yeast [15,21], as well as in other organisms [13,16-18,22,23]. A comprehensive review on NM theory and experimental approaches is currently available [24]. Knowledge of the NMs to which a given TF belongs facilitates the identification of downstream target gene clusters. In yeast, a genome-wide location analysis was carried out for 106 TFs and five NMs were considered significant: autoregulation, FFL, single input module, multi-input module and regulator cascade. The first four NMs are transcriptionally related, while the last one reflects the signalling pathway activities beyond transcriptional regulation.

In this study, we developed a computational framework that integrates information from time course gene expression experiment, genomic location analysis, binding site sequence, and GO category information to infer the relationship between TFs and their potential target genes based on known and predicted NMs. This was accomplished through a three-step approach outlined in the following. First, we applied cluster analysis of time course gene expression profiles to reduce dimensionality and use the GO category information to determine biologically meaningful clusters, upon which a model of the regulatory module is built. This step enables us to address the scalability problem that is faced by researchers in inferring TRNs from time course gene expression data with limited time points. Second, we trained support vector machines (SVMs) to classify TFs into different NMs based on their time course gene expression profiles, location analysis data, and target binding site sequences. The resulting SVM classifiers were utilized to predict NMs for TFs with unknown NMs. Finally, we used recurrent neural network (RNN) models that mimic the topology of NMs to identify gene clusters that may be regulated by a TF, thereby inferring the regulatory relationships between the TFs and gene clusters. A hybrid of genetic algorithm and particle swarm optimization (GA-PSO) methods was applied to train the RNN models. We tested the proposed computational framework using changes in gene expression associated with cell cycle progression in yeast [8], genomic location data [15], binding site sequences [25], and corresponding GO category information [26].

## Results

### Clustering genes into groups with enrichment for biological functions

**Figure 2:**
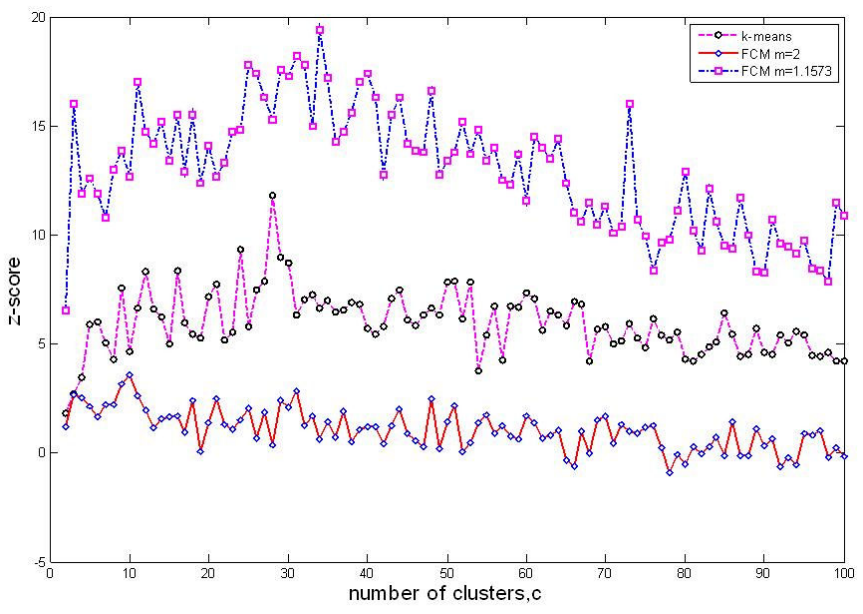
**Clustering results using k-means and FCM**. The cluster results from different cluster methods are compared using *z*-score, a measurement based on the mutual information between cluster membership and known gene attributes. Three clustering results are plotted: k-means clustering and FCM clustering with two *m *values (*m *is the fuzziness parameter): default value (*m *= 2) and optimal value (*m *= 1.1573). K-means outperforms FCM with default *m *value, whereas FCM with the optimal *m *value yields the highest *z*-score for cluster numbers ranging from 2 to 100. This demonstrates that FCM clustering with optimal *m *value has the potential to detect the underlying data structure with biological significance.

We selected 800 cell cycle-regulated genes and grouped them into clusters by fuzzy c-means (FCM), where genes with similar expression profiles are represented by a gene cluster or a metagene. The optimal cluster number is determined by the mutual information between gene clusters and their GO annotations (Figure 2). We compared the performance of FCM clustering with two different *m *values and the k-means clustering (Figure 2). The highest *z*-score (the maximal mutual information between gene clusters and their GO annotations) was obtained when the number of clusters is 34 by FCM clustering with *m *= 1.1573. We evaluated the resulting clusters through the gene set enrichment analysis (GSEA) method. Table 1 presents the 34 clusters and their corresponding enriched GO categories. All clusters except 10, 18, 21, 22, 25 and 26 are enriched in some GO categories. Details of all clusters are provided in Additional file 1. We used these clusters as metagenes in our subsequent analyses to reduce the search space for TF-target gene relationship inference.

**Table 1:** Gene set enrichment analysis (GSEA) for clusters generated by FCM with the optimal fuzziness value.

Cluster ID	# of genes in cluster	Enriched Functional Category	Total Genes in the category	Clustered Genes	P value
1	10	nucleosome	10	9	1.8E-27
		DNA binding	229	9	7.2E-13
2	25	steroid metabolic process	43	5	4.9E-07
		steroid biosynthetic process	32	4	5.8E-06
3	5	cytokinesis, completion of separation	11	5	4.9E-15
		cell separation during cytokinesis	13	5	1.4E-14
4	33	kinetochore	54	5	6.6E-06
		mitotic cell cycle	271	8	0.000047
5	16	cellular bud	150	6	9.2E-07
		cytoskeletal part	180	6	2.7E-06
6	29	dolichyl-phosphate-mannose-protein mannosyltransferase activity	7	3	2.8E-06
		protein amino acid O-linked glycosylation	16	3	0.000043
7	28	transporter activity	338	8	0.000063
		primary active transmembrane transporter activity	53	4	0.000071
8	37	microtubule	35	4	0.000042
		cytoplasmic microtubule	14	3	0.00006
9	11	cellular bud neck	115	5	6.9E-07
		site of polarized growth	152	5	2.7E-06
10	44	N/A	N/A	N/A	N/A
11	37	DNA helicase activity	75	14	1.1E-18
		mitotic recombination	41	7	2.1E-09
12	16	ribonucleoside-diphosphate reductase activity	4	2	0.000034
		cell cycle process	440	7	0.000043
13	28	plasma membrane	261	9	8.6E-07
		transmembrane transporter activity	246	7	0.000063
14	31	leading strand elongation	14	3	0.000035
		DNA replication	131	8	9.1E-08
		DNA metabolic process	710	15	1.1E-07
16	22	L-serine ammonia-lyase activity	3	2	0.000033
17	32	microtubule-based process	101	12	1.9E-14
		microtubule cytoskeleton	94	11	3.3E-13
18	15	N/A	N/A	N/A	N/A
19	20	cellular bud	150	6	4.1E-06
		site of polarized growth	152	5	0.000078
20	10	cell wall	114	7	5E-11
		glucanosyltransferase activity	6	2	0.000032
21	6	N/A	N/A	N/A	N/A
22	28	N/A	N/A	N/A	N/A
23	29	pentose transmembrane transporter activity	4	3	3.2E-07
		fructose transmembrane transporter activity	15	3	0.000035
24	35	chromosome	231	12	1.3E-09
		mitotic sister chromatid cohesion	22	5	8.4E-08
		DNA replication	131	8	3.4E-07
25	30	N/A	N/A	N/A	N/A
26	5	N/A	N/A	N/A	N/A
27	8	response to pheromone	94	7	8.6E-13
		conjugation with cellular fusion	119	7	4.7E-12
28	24	amine transmembrane transporter activity	16	4	2.6E-07
		polyamine transmembrane transporter activity	10	3	5.3E-06
29	17	sulfur metabolic process	67	11	7.3E-19
		methionine metabolic process	24	7	7E-14
30	30	cytoskeletal part	180	9	7E-08
		spindle	80	6	1.4E-06
31	14	energy reserve metabolic process	36	3	0.000055
		cellular carbohydrate metabolic process	213	5	0.000058
32	15	MCM complex	6	5	1.9E-13
		pre-replicative complex	15	6	2.4E-13
		DNA replication preinitiation complex	21	6	2.6E-12
33	6	cell wall	114	5	9.2E-09
		structural constituent of cell wall	19	3	4.3E-07
34	22	DNA-dependent DNA replication	97	10	5.8E-14
		replisome	15	5	4.8E-10

To evaluate whether the gene clusters are enriched in some known biological function or process, we performed GSEA for gene clusters generated from FCM clustering with m = 1.1573 and c = 34. The cut off P-value is 0.0001. The enriched function(s) are listed in the table. Significant function(s) are correlated with all gene clusters except cluster 10, 18, 21, 21, 25 and 26.

### Predicting NMs for TFs

**Figure 3:**
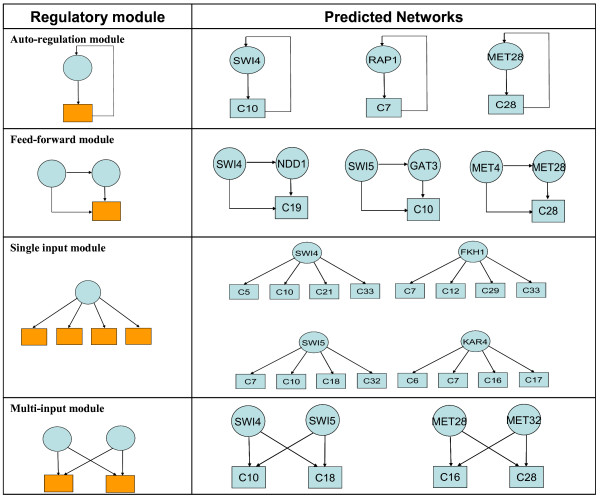
**Predicted NM from eight known cell cycle dependent TFs**. The left panel presents the four transcriptionally related NMs considered in this study. The right panel depicts inferred TF-target gene relationships for eight known cell cycle dependent TFs.

203 proteins were identified as DNA-binding transcriptional regulators in the yeast genome [27]. A genome-wide location analysis was carried out for 106 TFs and five NMs were considered significant (auto regulation, feed-forward, single input, multi-input, and regulator cascade). The first four NMs are transcriptionally related (shown in Figure 3, left panel), while the last one reflects the signaling pathway activities beyond transcriptional regulation (not shown). The 106 TFs include about 52% of the known TFs in the yeast genome.

Among the 800 cell cycle related genes, 85 have been identified to have TF-related functions based on their GO annotation. Out of these, 14 TFs have known NMs. A list of 85 TFs is presented in Additional file 2. We used data from 106 TFs to train SVM classifiers with time course gene expression profile and binding site sequence data as inputs to classify the TFs into four NMs. We retrieved the binding site sequence data for the TFs from the TRANSFAC database [28]. For TFs with unknown binding site sequences, we used the discovered binding site sequences described by Harbison et al. [27].

The trained SVM classifiers were evaluated and optimized using the LOOCV method. The final SVM classifiers were utilized to predict the NMs for 71 TFs with unknown NMs. Through the LOOCV method, we evaluated if both gene expression profile and binding site sequence information are needed in assigning TFs to NM categories. When we used gene expression profile alone as input to SVM, the average test error was 23.6%. After incorporating binding sequence data into the input data, the test error was reduced to 15.8% (Table 2). The increased performance implies that the encoded binding site sequence information is useful in predicting the critical TFs.

**Table 2:** Performance of SVM classifiers evaluated via LOOCV.

Input data	Auto regulation	Feed-forward loop	Single input	Multiple input	Average error
Gene expression data	3.7	48.1	17.6	24.8	23.6
Gene expression data and binding site sequence information	3.5	30.3	10.8	18.6	15.8

To evaluate the performance of the SVM classifiers, LOOCV was performed. To examine whether both gene expression data and binding site sequence information are needed in classifying TFs into different NM categories, we built SVM classifiers using only gene expression data. If only gene expression data are considered as input data, the average test error is 23.6%. After incorporating binding sequence data into the input data, test error has been reduced 15.8%. The increased performance implies that the encoded binding site sequence information is useful in predicting the biological roles TFs play.

### Inferring TF-target gene relationships in yeast

Recurrent neural network (RNN) models that mimic the topology of the known/predicted NMs were constructed to identify the relationships between TFs and putative gene clusters. The RNN models were trained to select for all 85 TFs the downstream targets from the 34 gene clusters.

Table 3 presents experimental results obtained for various numbers of generations that GA was used. The PSO generation for RNN is set to 1000 [29]. As illustrated in the table, the minimum value of RMSE decreases as the number of generations increases. The minimum RMSE for GA generations 600 and 800 are 0.077 and 0.075 respectively. In this study, we chose 600 for generations of GA. Our inference method mapped all 85 TFs to the target gene clusters and inferred the most likely NMs.

**Table 3:** The experimental results of GA-PSO with RNN.

GA generations	Average RMSE	Minimum RMSE
100	1.27	0.78
200	0.84	0.40
400	0.62	0.12
600	0.35	0.077
800	0.31	0.075

The average and least root mean square errors (RMSEs) obtained between the output of RNN and the measured expression profile for the gene clusters are shown as the number of GA generation is varied from 100 to 800.

We evaluated the predicted TF-target gene relationships for the following eight well known cell cycle related TFs: SWI4, SWI5, FKH1, NDD1, ACE2, KAR4, MET28 and RAP1. Among these, the first five have NM assignments, while the last three were assigned to different NMs by the SVM classifiers. Since the "true" gene regulatory network was not available, the accuracy of putative regulatory relationship was determined by searching known gene connections in databases. Based on the results of the NM module prediction, we collected literature evidences from SGD [30] and BIND [31] databases. We examined the inferred relationships for each of the eight TFs. An inferred relationship is assumed to be biologically significant if the TFs are correlated with the biological functions associated with the critical downstream cluster(s). Figure 3 lists the significant relationships; the eight TFs yielded an average precision of 82.9%. We calculated the precision as TP/(TP+FP), where TP and FP denote true positive and false positive, respectively. Network motifs for four of these TFs were identified in Chiang *et al*. [32] together with other four TFs. The eight TFs in [32] yielded an average precision of 80.1%.

## Discussion

The main goal of this study was to infer the components and underlying mechanism of gene regulation in yeast based on the combined constraints from multiple information sources. Our method effectively utilizes genomic location analysis for the establishment of NM for each TF. Target genes are grouped into biologically meaningful clusters and are represented by the average expression profiles of the genes in the cluster. Cluster analysis coupled with the idea of categorizing TFs into pre-defined NMs increased the robustness of our analysis not only in terms of obtaining meaningful modules, but also in terms of addressing the scalability problem. Some genes are very important in biological processes, thus are regulated through multiple pathways as shown by the presence of several distinct binding site sequences. Our proposed method allows the representation of a gene in different regulatory NMs since a TF can be assigned to more than one NM. This is different from previous approaches where only a single model is used for TRN inference [33,34].

Compared to previous methods that aimed at global TRN inference, the TF-target gene relationships inferred in this study are expected to correspond more closely to biologically meaningful regulatory systems and naturally lend themselves to optimum experimental design methods. For example, the results presented in Figure 3 can be verified from previous biological evidences. For example, FKH1 is a gene whose protein product is a fork head family protein with a role in the expression of G2/M phase genes. It negatively regulates transcriptional elongation, and regulates donor preference during switching. To further investigate the possibilities that the predicted downstream gene clusters are truly regulated by FKH1, we applied the motif discovery tool, WebMOTIFS [35] to find shared motifs in these gene clusters. The results revealed that a motif called Fork_head, GTAAACAA, is identified as the most significant motif among these gene clusters [36]. This finding strongly supports our NM inference results. The details of the binding site enrichment analysis (BSEA) results are shown in Additional file 3. Another example is the FFL involving SWI5, GAT3 and Gene Cluster 10. SWI5 has been identified as the upstream regulator of GAT3 [7,15,27]. Genes in cluster 10 are mostly involved in DNA helicase activity and mitotic recombination, both of which are important biological steps in the regulation of cell cycle. Although no biological evidences have shown that SWI5 and GAT3 are involved in these processes, there are significant numbers of genes in cluster 10 which are characterized (according to yeastract.com) as genes regulated by both TFs (24 for GAT3 and 23 for SWI5 out of 44 genes in cluster 10, respectively).

Compared to Chiang *et al*. [32], the first improvement of our approach is that instead of predicting the TF and individual downstream genes, we group genes into biologically functional clusters and discover the relationships between TFs and gene clusters. Through clustering, we were able to integrate the GO information, reduce the computational complexity, and established insights into new interactions. If a gene cluster is involved in the NM of one TF, and most genes have evidence that they are regulated by this TF, it is most likely that the genes left in this cluster are under the regulatory control of the TF. Furthermore, the intermediate result analysis such as GSEA and motif discovery analysis employed in our method ensure that every step in the data integration contributes to the final NM inference.

## Conclusion

Reconstruction of TRNs is one of the major challenges in post genomic era. The study presented here addressed two important issues in TRN inference: (1) the development of analysis methods that utilizes multiple types of data and (2) network analysis on the NM level. A data integration approach is proposed to effectively infer the underlying mechanism and pattern of gene regulation using yeast as model on the basis of combined constraints arising from multiple biological data sources, including time course gene expression data, location analysis data, binding site sequence data and GO category information. This computational framework allows us to fully exploit the partial constraints that can be inferred from each data source. First, to reduce the inference dimensionalities, the genes are grouped into clusters by FCM, where the optimal fuzziness value is determined by statistical properties of gene expression data and the optimal cluster number is identified by integrating the GO category information. Then, the known NM information from location data analysis together with the binding site information is used to train SVM classifiers. TFs without NM assignment are predicted by the classifiers. LOOCV is used to build the SVM classifiers with high confidence. Once the NM(s) for a TF is identified, the hybrid GA-PSO algorithm is applied to search for target gene clusters that may be regulated by the TF. This search is guided by the successful training of a RNN model that mimics the regulatory NM(s) assigned to the TF. This has been demonstrated on eight well-studied yeast cell cycle dependent TFs. The upstream BSEA indicates that the proposed method has the potential to identify the underlying regulatory relationships between TFs and their downstream genes on the NM level. We conducted a thorough evaluation of our approach by applying it to a well studied process in yeast (regulation of cell cycle progression). Although we limited our analysis to gene regulatory program at the transcriptional level, we believe that our model is expandable to other biological network inference as more types of high-through data become available such as protein-protein interaction data (yeast two-hybrid) and *in vivo *(yeast one-hybrid) and *in vitro *(chromatin immunoprecipitation) protein-DNA interaction data. We anticipate that this approach will serve as a novel method for analyzing multi-source data on the NM level.

## Methods

### Approach overview

The data sources used in this study involve two information levels: (1) The location analysis data, binding site sequences, and GO category information characterize the physical interactions at factor-gene binding level; (2) The time course expression data characterize the functional interactions at transcriptional regulation level. The goal is to discern dependencies between the gene expression profiles and the physical (molecular interaction) mechanisms revealed by complementary data sources (e.g., location data and binding site sequences).

The genome-wide location analysis is a genomic scale assay [5] measuring the *in vivo *abundance of TFs that bind to intergenic regions of the DNA. Unlike the expression data, location analysis provides direct evidence about the physical processes underlying gene regulation. Available data from location analysis experiments of 106 TFs, representing ~52% of the total TFs encoded by yeast genome were used in this study to determine transcriptional NMs.

The DNA sequence motifs that define transcription factor binding sites (TFBSs), were extracted from TRANSFAC database [28]. Additional information for other TFs were obtained from recent data as described by Harbison *et al*. [27].

GO information was used as the source of gene annotations from already validated biological evidences [26]. Three GO categories (biological process, molecular function, and cellular component) were used as a basis to determine/evaluate the optimal number of gene clusters.

Gene expression profiling represents a high-throughput data source, where expression levels for thousands of genes are measured simultaneously. Models such as Bayesian networks [37] or probabilistic relational models [11] have been used to capture the interactions among the measured expression levels. The limited number of time points and the large number of genes present a challenge in inferring TRNs from time course gene expression data. The yeast (*S. cerevisiae*) cell cycle data are based on the changes in gene expression in terms of transcript abundance at six stages (cln3, clb2, alpha, cdc15, cdc28, and elu) [8]. A total of 800 genes were identified as cell cycle-regulated based on cluster analysis [8]. In this study, we chose the cdc15 expression data set for 800 genes, because this set has the largest number of time points (24).

**Figure 4:**
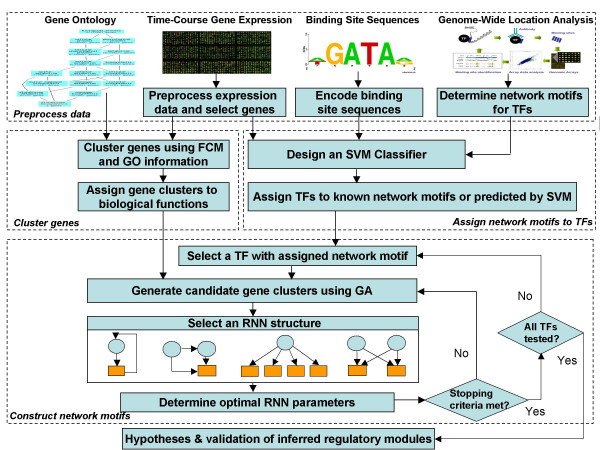
**The proposed computational framework**. The framework of the proposed method is composed of three parts. First, gene expression profiles are clustered into biologically meaningful groups by FCM; GO category information of genes is used to determine the optimal cluster number. To evaluate the gene clusters, gene set enrichment analysis (GSEA) is performed on the optimal clusters. This analysis revealed that 28 out of 34 optimal clusters were enriched in certain biological categories (P-value < 0.001) (Table 1). In NM assignment part, SVM classifiers are built to classify TFs into known NM categories. For a given TF, its time course gene expression profile and binding site sequences are used as inputs to SVM classifiers to predict its corresponding NM(s). Positive training data sets include TFs with known NMs from location data analysis. Negative training data sets include TFs randomly chosen from TF pools (same size as positive ones). After the gene clusters are formed and TFs are assigned to NM categories, the relationships between TFs and gene clusters are inferred by training recurrent neural networks (RNNs) that mimic the topologies of the NMs that TFs are assigned to. Since the NM inference only includes small number of TFs and gene clusters, the computational complexity is reduced compared to the global TRN inference problem (inferring TRN on gene level by including all genes in one data set). Finally, the inferred TF-target gene relationships are validated by BSEA and literature results.

Our proposed computational framework is illustrated in Figure 4. Besides data pre-processing, there are three successive steps involved in this framework. The first step is gene clustering, where features with similar profiles are grouped together as a metagene (a gene cluster) to address the scalability problem [38]. The basic assumption is that a cluster of co-regulated genes share common TFs [39]. To evaluate the clustering performance, GO categories are utilized to determine the number of clusters and annotate gene clusters. Since each cluster mainly represents one function or process category (evaluated by FuncAssociate [40]), the regulation network between a TF and a gene cluster implies that the TF can regulate a group of genes with similar or related functions [41]. In the second step, an NM is assigned to a TF, wherein NMs are used instead of global TRN inference to reduce the complexity of the inference problem by building SVM classifiers that assign NM(s) to each TF. TFs with known NMs are used as a training set [15]. The trained SVM classifiers are applied to predict NMs for TFs with unknown NMs. To evaluate the classifier performance, leave-one-out cross-validation (LOOCV) is applied. In the third step, for each TF with either known or predicted NM(s), GA generates candidate gene clusters that may be regulated by the TF according to the NM. A RNN is trained to mimic the known or predicted NM. PSO optimizes the parameters of the RNN to minimize the root mean squared error (RMSE) between the output of the RNN and the target gene cluster's average expression profiles. The RMSE is returned to GA to produce the next generation of candidate gene clusters. The optimization is continued until a pre-specified maximum number of iterations or a pre-specified minimum RMSE is reached. The above procedure is repeated for all TFs. Known biological knowledge from databases is used to evaluate the predicted results.

Table 4 summarizes the inputs and outputs of each step involved in our proposed computational framework. The steps are elaborated in more details in the following sub-sections.

**Table 4:** Inputs and outputs of the proposed three-step approach

Steps	Inputs	Output(s)	Figure
Cluster genes into biologically relevant groups	- Gene expression profiles	Cluster centers/metagenes	Fig. 6
	- GO category information		
Categorize TFs into different NMs	Gene expression profile of a TF	Predicted NM(s) for the TF	Fig. 7
	- Encoded binding site sequences for the TF		
Infer NM-based TF-target relationship via RNN	- Gene expression profile of a TF	Identified cluster centers in the known or predicted NMs (i.e., identified TF-target gene relationship).	Fig. 8
	- Gene expression profiles of cluster centers (metagenes)		
	- Known or predicted NM(s) of the TF		

### Data preprocessing

From the time course gene expression data, 800 genes are identified as being cell cycle-regulated based on an analysis that combines a Fourier algorithm and a correlation algorithm [8]. These genes are functionally annotated based on information from GO. Missing values in the data are imputed using K nearest neighbour (KNN) imputation [42]. Following that, the expression profile of each gene was standardized between 0 and 1.

**Figure 5:**
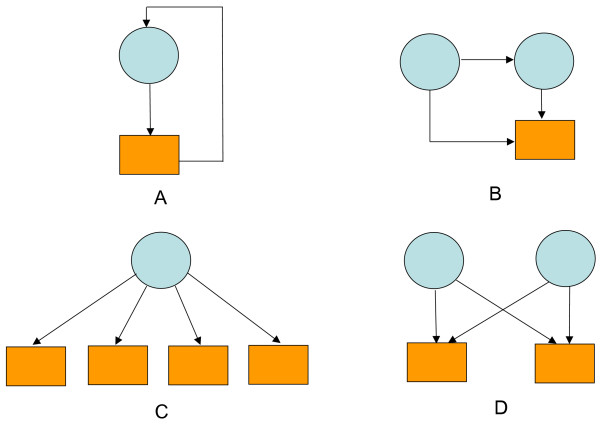
**Four transcriptional network motifs in yeast**. Four NMs are considered in this study: (**A**) auto-regulatory motif; (**B**) feed-forward loop; (**C**) single input module; and (**D**) multi-input module. Circle denotes TF and square denotes gene cluster.

Known NMs are extracted from location analysis data [15]. By specifying a threshold value (e.g. P-value < 0.001) that represents the confidence that a given factor binds to the corresponding intergenic region, the location data can be viewed as a combination of four NMs (Figure 5). Nucleic acids are encoded into numeric values (Table 5) so that the binding site sequence information derived from TRANSFAC database can be used as input to SVM classifiers for NM prediction. Although the numerical values assigned to nucleic acids do not carry any biological meaning, they were used in our analysis for the purpose of implementing the SVM classifier.

**Table 5:** Encoding of nucleic acids by numeric values.

Nucleic acid	A	C	G	T	U	R	Y	M
Numeric code	0.05	0.1	0.15	0.2	0.25	0.3	0.35	0.4
Nucleic acid	K	W	S	B	D	H	V	N
Numeric code	0.45	0.5	0.55	0.6	0.65	0.7	0.75	0.8

To incorporate the binding site sequence information into the NM inference, we encode the nucleic acid sequence by numerical values according to the nominal transitions in the table.

### Cluster genes into biologically relevant groups

We use cluster analysis to assign genes into functional groups and use the resulting cluster nodes as metagenes. Clustering is a widely used technique in microarray data analysis. An underlying assumption is that genes with similar expression profiles are more likely to have similar biological functions [43]. Common clustering algorithms such as hierarchical clustering, k-means clustering, and self-organized maps have been used to analyze gene expression data [9,44,45]. These are called hard clustering because each gene is assigned to exactly one cluster. Microarray data involve substantial amount of noise due to biological and experimental factors. In this study, we utilize a soft clustering approach using FCM, which has been demonstrated to be resilient to noise; genes with high membership values cluster together in spite of the noise in the gene expression data [46].

**Figure 6:**
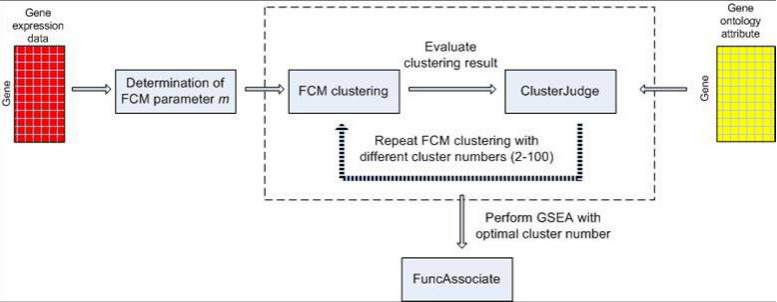
**The FCM clustering scheme**. The scheme illustrates the process to group genes into biologically meaningful clusters. The gene expression data are first utilized to find the optimal *m *value for FCM clustering. With the optimal *m *value, FCM clustering is performed on gene expression data for cluster numbers ranging from 2 to 100. The cluster results were then evaluated by using the ClusterJudge software. The cluster number with the largest *z*-score is chosen as optimal. The GSEA (FuncAssociate) is performed to evaluate the gene clusters formed using the optimal cluster number.

The detailed clustering scheme is shown in Figure 6. The fuzziness parameter *m*, and the cluster number *c *need to be determined in FCM clustering. The optimal value for *m *varies widely from one data set to another. An empirical method [46] is applied to determine an adequate value for *m *based on the distribution of distances between genes. The optimal cluster *c *is evaluated by the ClusterJudge software [43], which estimates the optimal cluster number using a figure of merit based on the mutual information between cluster membership and known gene attributes in GO database. GO database contains three categories: molecular function, biological process, and cellular components, to describe attributes of gene products or gene product groups [26]. All the gene attributes in three categories are filtered based on the following criteria: (1) they are as independent as possible (one of any attribute pair that has a pair-wise uncertainty coefficient U > 0.8 is removed, U = MI/MI_max_, where MI denotes the mutual information between two gene attributes and MI_max _is the maximum MI among all gene attributes); (2) they are shared among 10~200 genes. Those passing the filtering are used for selecting the optimal cluster number. ClusterJudge calculates z-score to evaluate the gene attributes that genes belong to, in contrast to other data-driven approaches such as Xie-Beni index [47], gap statistic [48], and adoptive double self organizing map [49] that do not involve biological evaluation.

To characterize the optimal gene clusters, we utilize FuncAssociate [40] that determines GO terms that are over-represented among the genes associated with a given cluster relative to what would be expected for randomly chosen sets of genes of the same size. FuncAssociate computes a one-tailed Fisher's exact test whose categories are "belongs/does not belong to cluster" and "is annotated/is not annotated with GO term". This computation is performed for all the GO terms for which annotations are available, and the P-values obtained are corrected for multiple hypotheses by comparing raw P-values against those obtained from 1000 simulated runs using randomized queries (resampling), as described in detail in Berriz et al. [40]. The definition of the 'universe' of all genes used by FuncAssociate corresponds to the set of all genes used in FCM clustering. Once the cluster analysis is completed, a set of genes in a cluster is considered as a metagene in the subsequent analyses.

### Categorize TFs into different NMs

Since GO has the most detailed gene annotation, we first search for genes with GO functional annotation terms related to transcription such as "transcriptional regulator activity", "DNA binding", etc. These genes are treated as potential TFs and also verified by comparison with the TRANSFAC database. The TF list in TRANSFAC only contains known TFs. The detailed annotation of GO provides a larger list containing not only the confirmed but also the potential TFs. These TFs are assigned to different kinds of motifs by their characteristic of regulation functions. This is based on the assumption that some TFs play crucial roles in some specific motifs. Unlike to most previous TRN inference approaches, where a single large network is sought, our method focuses on inferring target cluster gene(s) regulated by a particular TF. This is accomplished by assigning likely NM(s) to each TF based on prior biological knowledge collected from literatures that report on results from traditional experiments or large-scale genomic location analysis data [15].

**Figure 7:**
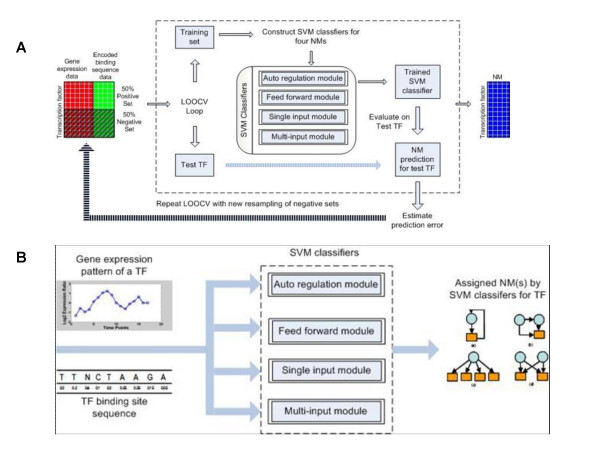
**SVM classifiers that predict the network motif for a TF on the basis of its binding site sequence and its time course gene expression profile**. The figure shows the scheme for classifying TFs into four NMs. Since one TF can be assigned to more than one NM, one SVM classifier is built for each NM assignment (four classifiers in the SVM classifier block). We illuatrate the process in: (**A**) training phase, where the TFs with known NMs are used to train SVM classifiers, (**B**) operation phase, where unknown NMs are predicted by the trained SVM classifiers based on expression profile and binding site of a TF. In the training phase (**A**), a data set that consists of expression profile and binding site sequences is constructed for each classifier. The data set has positives (TFs with known NMs), and negatives are TFs to which randomly chosen NMs are assigned (equal in size to the positive set). The data set is used to suit the SVM classifiers. The classifiers are evaluated through the LOOCV approach (dashed box) to estimate their prediction errors. In the operation phase (**B**), the expression profile and binding site of a TF with unknown NM assignments are used as inputs to the SVM classifiers trained in (**A**). The classifiers predict the NM(s) for the TF.

Since only a fraction of TFs have known NMs, we build SVMs to map the relationship among a gene expression profile of a TF, its binding site sequence data, and its NM(s). The nucleic acids in the binding site sequence data are encoded into numeric values (Table 5) before presenting them to the SVM classifiers. Figures 7A and 7B depict the SVM training and operation phases, respectively. In the training phase, a data set that consists of expression profile and binding site sequences is constructed for each classifier. The data set has positives (TFs with known NMs) and negatives (TFs to which randomly chosen NMs are assigned) with equal proportions. This data set is used to train the SVM classifiers. The classifiers are evaluated through the LOOCV approach to estimate their prediction errors. In the operation phase, the expression profile and the binding site sequence of a TF with unknown NM assignments are used as inputs to the trained SVM classifiers to predict the NM(s) for the TF. The figures show four NM modules that are used in this study (auto regulation, feed-forward, single input, and multi-input). Since a TF can be assigned to more than one NM, a binary SVM that can handle only two cases is not sufficient. Thus, as illustrated in figures, we use multiple binary SVM classifiers, each responsible for one NM. Each SVM is trained to determine whether a TF can be assigned to the NM. We input the expression profile and binding site sequence into each of the four trained SVM classifiers to obtain a yes or no answer. The classifiers are evaluated using LOOCV. We outline below the steps involved:

1. Assemble positive set from genome-wide location data. Sample *n *TFs randomly from the whole TF set to construct the negative set (*n *= number of TFs in positive set).

2. Leave the first TF out as a test TF; the remaining TFs serve as a training set.

3. Build SVM classifiers using the training set.

4. Use trained SVM classifiers to determine the NM(s) for the TF left out in Step 2.

5. Replace the left out TF and leave the next TF out as a test TF.

6. Repeat Steps 3–5 until each TF is used as a test TF.

7. Summarize the prediction error for the left out TFs.

8. Repeat steps 1–7 100 times.

9. Calculate the mean of the predicted error in 100 runs.

The final SVM classifiers are trained by using all TFs with known NMs as a positive set and an equal number of randomly selected TFs as a negative set. The NM(s) for a TF with unknown NM(s) is determined using these classifiers.

### Infer NM-based TF-target relationship via RNN

**Figure 8:**
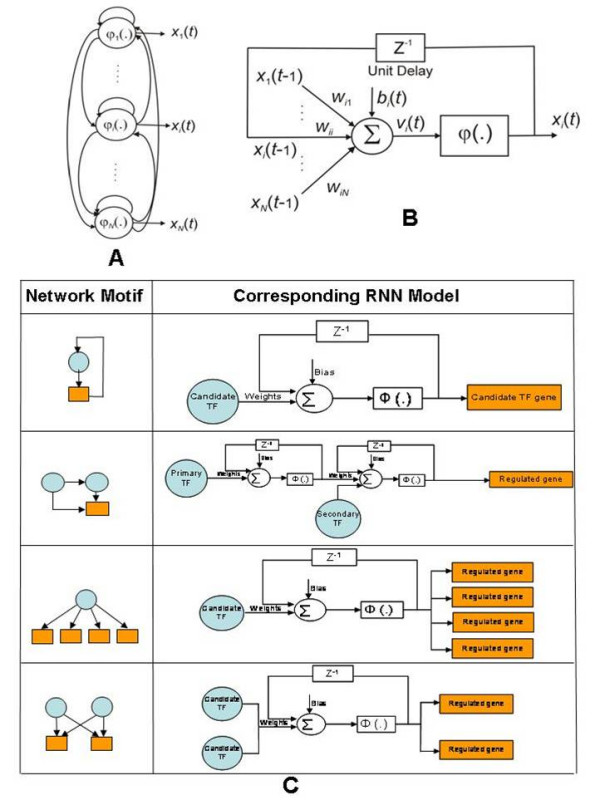
**The RNN models for NMs**. **A**: RNN model, where the output of each neuron is fed back to its input after a unit delay and is connected to other neurons. **B**: Details of a single recurrent neuron. **C**: RNN models mimicking the topologies of the four NMs shown in Figure 5. Z^-1 ^denotes a unit delay and Φ(.) is a logistic sigmoid activation function.

After deciding the NM(s) for all TFs, we construct a model of the NM for each TF via a RNN, whose topology mimics the NM that the TF is known or predicted to exhibit. Due to its capability to capture the nonlinear properties and dynamic relationships, RNNs have been previously applied for GRN inference [33,50,51]. For each of the four NMs in Figure 5, a suitable RNN can be built (Figure 8). As shown in Figure 8C, each RNN has an architectural layout that mimics the corresponding NM. The rationale for using RNNs to model gene NMs emanates from their ability to learn from data and to simulate gene regulation through the formulation shown in Eq. (1) [52,53]:

(1) $$\frac{dx_{i}(t)}{dt}=-\tau x_{i}(t)+\phi \left(\sum_{j=1}^{N}w_{ij}x_{j}(t)+b_{i}\right)$$

where *x*_*i *_is the gene expression level of the *i*th gene (1 ≤ *i *≤ *N*, *N *is the number of genes in the model), *φ*(.) is a activation function introduces nonlinearity to the model (e.g. sigmoid function), *w*_*ij*_represents the effect of *j*th gene on the *i*th gene, *b*_*i *_denotes the bias for the *i*th term, and *τ *is the decay rate parameter. A negative value of *w*_*ij *_represents the inhibition of the *j*th gene on the *i*th gene, whereas a positive value of *w*_*ij *_represents the activation control of the *j*th gene on the *i*th gene. If *w*_*ij *_is zero, then it means that *j*th gene has no influence on the *i*th gene.

The discrete form of Eq. (1) can written as

(2) $$x_{i}(t+\Delta t)=(1-\tau \Delta t)x_{i}(t)+\Delta t\phi \left(\sum_{j=1}^{N}w_{ij}x_{j}(t)+b_{i}\right)$$

Figures 8A and 8B show the architecture of a RNN that can simulate the mathematical relationship in Eq. (2). As illustrated in Figures 8A and 8B, the output of each neuron is fed back to its input after a unit delay and is connected to other neurons [51]. It can be used as a simple form of a NM, where each entity (e.g. TF or gene cluster) in the network is considered as a neuron. The RNN can model not only the interactions between entities but also entity self-regulation. In this study, we consider four RNN models (Figure 8C), each of which has an architectural layout that mimics the corresponding NM in Figure 5.

Training the RNNs involves determining the optimal weights *w*_*ij *_and bias *b*_*i*_. As a cost function, we use the RMSE between the expected output and the network output across time (from the initial time point 0 to the final time point T) and across neurons in the network. The cost function can be written as:

(3) $$E(\vec{w})=\sqrt{\frac{1}{TN}\sum_{t=0}^{T}\sum_{i=1}^{N}{\left[x_{i}(t)-{\hat{x}}_{i}(t)\right]}^{2}}$$

where *x*_*i*_(*t*) and ${\hat{x}}_{i}(t)$ are the true and predicted values (expression levels) for the *i*th neuron (entity) at time *t*. The goal is to determine the structure and weights of a RNN that minimize this cost function.

A hybrid of GA and PSO methods (GA-PSO) is applied to determine the gene clusters that may be regulated by each TF. GA generates candidate gene clusters, while the PSO algorithm determines the parameters of a given RNN represented by a weight vector $\vec{w}$. The RMSE between the RNN output and the measured expression profile is returned to GA as a fitness function and to guide the selection of target genes through reproduction, cross-over, and mutation over hundreds of generations. The stopping criteria are pre-specified minimum RMSE and maximum number of generations. The GA-PSO algorithm is run for each TF to train a RNN that has the architecture mimicking the known NM(s) for the TF or the NM(s) predicted by the SVMs. Thus, f or a given TF (input), the following steps are carried out to identify its likely downstream gene clusters (output) based on known or predicted NM(s):

1. Assign the NM to the TF it belongs to. If the NM is unknown, use SVM to predict the NM(s).

2. Use the following GA-PSO algorithm to build a RNN model that mimics the NM to identify the downstream gene clusters.

2.1. Generate combinations of *M *gene clusters to represent the target genes that may be regulated by the TF. Each combination is a vector/chromosome. The initial set of combinations is composed of the initial population of chromosomes.

2.2. Use the PSO algorithm to train a RNN model for each chromosome, where the input is the TF and the outputs are gene clusters. The goal is to determine the optimized parameters of the RNN that maps the measured expression profiles of the TF to the gene clusters.

2.3. For each chromosome, calculate the RMSE between the predicted output of the RNN and measured expression profiles for the target gene clusters.

2.4. Apply GA operators (reproduction, cross-over, mutation) based on the RMSE calculated in Step 2.3 as a fitness value. This will generate new vectors/chromosomes altering the choice of output gene cluster combinations.

2.5. Repeat steps 2.1 – 2.4 until stop criteria are met. The stopping criteria are numbers of generations or minimum RMSE, depending on which one is met first.

2.6. Repeat Steps 2.1 – 2.5 for each NM the TF is assigned to.

3. Repeat Steps 1 and 2 for each TF.

When the process is completed, regulatory NMs are constructed between TFs and their regulated gene clusters.

We used the OSU SVM Support Vector Machine Toolbox [54] for implementation of SVMs. The Genetic Algorithm and Direct Search Toolbox (Mathworks, Natick, MA) and the PSOt Toolbox [55] were utilized for implementation of GA and PSO, respectively. The parameter settings of GA and PSO are shown in Table 6.

**Table 6:** GA and PSO parameter settings. The table presents the GA and PSO parameter settings used in this study.

PSO		GA	
	
Parameter	Value	Parameter	Value
Search space range	[-5,5]	Crossover	One point
Acceleration constants *c*_1_	2.05	Mutation rate	0.05, random
Acceleration constants *c*_2_	2.05	Selection	Roulette Wheel
Size of swarm	50–150	Population size	50–150

## List of abbreviations

Transcriptional regulatory network: TRN; network motif: NM; transcription factor: TF; fuzzy c-means: FCM; gene set enrichment analysis: GSEA; binding site enrichment analysis: BSEA; support vector machine: SVM; leave-one-out cross-validation: LOOCV; recurrent neural network: RNN; genetic algorithm: GA; particle swarm optimization: PSO; gene ontology: GO; root mean squared error: RMSE; feed-forward loop: FFL; transcription factor binding site: TFBS; transcriptional start site: TSS.

## Authors' contributions

YZ and HWR designed the computational approach, wrote the code, analyzed the experimental results and drafted the manuscript. All authors read and approved the final manuscript.

## Supplementary Material

Additional file 1 Gene clusters obtained by using the FCM clustering algorithm. The data provided present the cluster membership for each of the 800 genes considered in this study. Click here for file

Additional file 2 Potential TFs among yeast cell cycle related genes. The data provided present TFs we identified from 800 cell cycle related genes based on their GO annotations. The genes annotated with terms related to transcription activities and DNA binding are considered as potential TFs. Click here for file

Additional file 3 Predicted motifs for gene clusters. The data provided present the top three enriched motifs for each gene cluster identified in this study. The motifs are predicted through promoter sequence analysis of the gene clusters using WebMOTIFS . This information helps in the validation of the NM prediction results. For example, the predicted downstream gene clusters of FKH1 all have a motif called Fork_head, GTAAACAA, in their promoter regions. This suggests that our NM inference strategy has the capability to identify the downstream target genes for TFs based on their NM assignment. The motif is annotated in Pfam database . Click here for file
