# Supplementary material for: Network motif-based identification of transcription factor-target gene relationships by integrating multi-source biological data
Source: BMC Bioinformatics. 2008 Apr 21;9:203. doi: 10.1186/1471-2105-9-203 (PMC2386822; doi:10.1186/1471-2105-9-203)
Supplement: Additional file 1 — Gene clusters obtained by using the FCM clustering algorithm. The data provided present the cluster membership for each of the 800 genes considered in this study. [file 1471-2105-9-203-S1.pdf]

Additional file 1 – Gene clusters obtained by using the FCM clustering algorithm

We present cluster membership for each of the 800 genes considered in this study.

ORF	Cluster #	ORF	Cluster #	ORF	Cluster #	ORF	Cluster #	ORF	Cluster #
YBL002W	0	YBR243C	7	YGL008C	12	YBR158W	18	YNR066C	24
YBL003C	0	YCL062W	7	YGL255W	12	YDL117W	18	YOL058W	24
YBR009C	0	YCL063W	7	YGR240C	12	YDL179W	18	YOL088C	24
YBR010W	0	YDR130C	7	YGR279C	12	YGL055W	18	YOR250C	24
YDL055C	0	YDR150W	7	YIL011W	12	YGR041W	18	YOR332W	24
YDR224C	0	YDR261C	7	YLR084C	12	YGR044C	18	YOR391C	24
YDR225W	0	YDR302W	7	YLR100W	12	YGR086C	18	YPL025C	24
YNL030W	0	YDR346C	7	YLR169W	12	YIL009W	18	YPL054W	24
YNL031C	0	YER032W	7	YLR353W	12	YIL104C	18	YPR107C	24
YPL127C	0	YGL101W	7	YML052W	12	YJL078C	18	YPR155C	24
YBR094W	1	YGL216W	7	YML064C	12	YJL217W	18	YBR054W	25
YDR089W	1	YGR138C	7	YML072C	12	YKL116C	18	YBR092C	25
YEL025C	1	YHR098C	7	YML116W	12	YLR079W	18	YDR033W	25
YER145C	1	YHR108W	7	YNL172W	12	YLR194C	18	YNL160W	25
YGR176W	1	YHR135C	7	YOL030W	12	YNL046W	18	YPR149W	25
YGR177C	1	YHR205W	7	YOR153W	12	YNL078W	18	YCL027W	26
YGR259C	1	YKL004W	7	YOR298W	12	YNL192W	18	YCL055W	26
YGR260W	1	YKL048C	7	YPR128C	12	YOR263C	18	YGL089C	26
YHL040C	1	YKL096W-A	7	YPR138C	12	YOR264W	18	YGL090W	26
YHR151C	1	YKR037C	7	YBL061C	13	YPL158C	18	YJR004C	26
YIL094C	1	YLR180W	7	YBR087W	13	YDR451C	19	YKL177W	26
YIL119C	1	YLR209C	7	YDL103C	13	YFL037W	19	YKL178C	26
YJR003C	1	YLR437C	7	YDR053W	13	YIL123W	19	YLR452C	26
YKR079C	1	YML065W	7	YDR190C	13	YIL129C	19	YNR044W	26
YLR056W	1	YML125C	7	YDR279W	13	YJL158C	19	YBL030C	27
YLR214W	1	YMR002W	7	YDR440W	13	YKL096W	19	YCL025C	27
YLR413W	1	YMR163C	7	YEL076C-A	13	YLR300W	19	YCLX09W	27
YLR438W	1	YMR198W	7	YER149C	13	YMR215W	19	YDR380W	27
YML123C	1	YNL043C	7	YGL185C	13	YMR307W	19	YHR137W	27
YMR015C	1	YNL216W	7	YGR042W	13	YOR247W	19	YJR048W	27
YMR202W	1	YOL012C	7	YGR234W	13	YOR248W	19	YKL035W	27
YNL111C	1	YOL112W	7	YGR276C	13	YBR204C	20	YKL043W	27
YNR050C	1	YOR323C	7	YHR106W	13	YCR018C	20	YKR039W	27
YOR383C	1	YOR324C	7	YHR126C	13	YGR238C	20	YKR046C	27
YPL036W	1	YOR337W	7	YIL076W	13	YLR347C	20	YLL028W	27
YPL075W	1	YPL128C	7	YJR043C	13	YNL326C	20	YLR058C	27
YPL111W	1	YPR034W	7	YKL172W	13	YOR023C	20	YLR142W	27
YER124C	2	YPR111W	7	YKR091W	13	YAR003W	21	YML120C	27
YGL028C	2	YBR038W	8	YLR135W	13	YDL010W	21	YMR058W	27
YHR143W	2	YDR146C	8	YML020W	13	YDR307W	21	YMR145C	27
YLR286C	2	YGL021W	8	YMR246W	13	YDR481C	21	YMR189W	27
YNR067C	2	YGR108W	8	YNL231C	13	YEL064C	21	YNL037C	27
YBR133C	3	YLR190W	8	YOL094C	13	YFL060C	21	YOL119C	27
YDR247W	3	YML119W	8	YOR075W	13	YGL060W	21	YOR256C	27

ORF	Cluster #	ORF	Cluster #	ORF	Cluster #	ORF	Cluster #	ORF	Cluster #
YEL017W	3	YMR001C	8	YOR115C	13	YGL163C	21	YOR273C	27
YER018C	3	YMR032W	8	YOR242C	13	YGL207W	21	YPL021W	27
YGL125W	3	YNL058C	8	YOR283W	13	YGR153W	21	YPL061W	27
YGR113W	3	YPR119W	8	YOR307C	13	YHR123W	21	YPL250C	27
YHR086W	3	YPR156C	8	YOR308C	13	YHR154W	21	YPL265W	27
YHR146W	3	YBL052C	9	YOR355W	13	YHR159W	21	YAL067C	28
YHR178W	3	YBR242W	9	YPL232W	13	YJL015C	21	YER042W	28
YIL050W	3	YCRX05W	9	YBR073W	14	YJL072C	21	YER091C	28
YIL131C	3	YDL048C	9	YCR065W	14	YJR054W	21	YFR030W	28
YIL144W	3	YDL180W	9	YDL018C	14	YJR155W	21	YGL184C	28
YIR010W	3	YDR011W	9	YDL101C	14	YKL089W	21	YGR055W	28
YJL119C	3	YDR029W	9	YDL163W	14	YKL182W	21	YIR017C	28
YJL134W	3	YDR149C	9	YDL164C	14	YLR050C	21	YJL060W	28
YJL137C	3	YFR039C	9	YDR400W	14	YLR151C	21	YJR010W	28
YJR001W	3	YGL195W	9	YDR507C	14	YLR233C	21	YJR137C	28
YKL069W	3	YGL209W	9	YHR110W	14	YML021C	21	YKL001C	28
YKR010C	3	YGR035C	9	YHR149C	14	YML133C	21	YKR069W	28
YKR041W	3	YHR029C	9	YIL141W	14	YNL181W	21	YLL061W	28
YLL032C	3	YIL056W	9	YJL073W	14	YOL034W	21	YLL062C	28
YLR288C	3	YIL122W	9	YJL074C	14	YOR176W	21	YLR302C	28
YLR455W	3	YJL099W	9	YJL187C	14	YPL014W	21	YLR303W	28
YMR003W	3	YJR110W	9	YKL113C	14	YPL057C	21	YNL276C	28
YMR295C	3	YKL130C	9	YLL002W	14	YBR108W	22	YPL274W	28
YNL176C	3	YKL183W	9	YLL022C	14	YBR256C	22	YPR167C	28
YNL197C	3	YKR021W	9	YLR103C	14	YBR273C	22	YBL009W	29
YOR073W	3	YLR034C	9	YLR313C	14	YDL089W	22	YBL063W	29
YOR083W	3	YLR057W	9	YLR383W	14	YDL169C	22	YDR113C	29
YOR372C	3	YLR098C	9	YML060W	14	YDR085C	22	YDR297W	29
YPL116W	3	YML035C-A	9	YMR078C	14	YDR342C	22	YDR355C	29
YPL253C	3	YMR183C	9	YNL072W	14	YDR493W	22	YEL042W	29
YPL264C	3	YMR254C	9	YNL102W	14	YEL060C	22	YEL061C	29
YPL269W	3	YNL056W	9	YNL233W	14	YEL068C	22	YER003C	29
YCL012W	4	YNL171C	9	YNL300W	14	YGR219W	22	YGL225W	29
YCL013W	4	YOL014W	9	YNL312W	14	YHR067W	22	YGR014W	29
YCL014W	4	YOL069W	9	YOR074C	14	YHR094C	22	YGR099W	29
YCR024C-A	4	YOL114C	9	YPL208W	14	YHR113W	22	YHR061C	29
YHL028W	4	YOR058C	9	YPL221W	14	YIL114C	22	YHR173C	29
YHR023W	4	YOR104W	9	YPR135W	14	YIR036C	22	YJL092W	29
YIL106W	4	YOR105W	9	YPR174C	14	YJL044C	22	YJL118W	29
YIL158W	4	YOR129C	9	YBR157C	15	YJL067W	22	YJL201W	29
YJL051W	4	YOR152C	9	YCL065W	15	YJL196C	22	YKL008C	29
YJR092W	4	YOR235W	9	YCL066W	15	YKL104C	22	YLL012W	29
YLR131C	4	YOR320C	9	YCR040W	15	YKL151C	22	YLR045C	29
YML033W	4	YPL058C	9	YCR041W	15	YLR231C	22	YLR342W	29
YML034W	4	YPL133C	9	YDL037C	15	YLR273C	22	YLR372W	29
YML058W	4	YPR013C	9	YDR461W	15	YML110C	22	YLR373C	29
YOR025W	4	YPR014C	9	YER150W	15	YMR011W	22	YLR380W	29
YOR315W	4	YPR045C	9	YFL026W	15	YNL015W	22	YMR144W	29
YPL141C	4	YPR157W	9	YFL044C	15	YNL208W	22	YNL126W	29
YPL155C	4	YBL111C	10	YGL162W	15	YOL016C	22	YNL166C	29

ORF	Cluster #	ORF	Cluster #	ORF	Cluster #	ORF	Cluster #	ORF	Cluster #
YPL242C	4	YBL112C	10	YGR146C	15	YOR018W	22	YNL283C	29
YDL093W	5	YBL113C	10	YGR284C	15	YOR052C	22	YNR009W	29
YDL095W	5	YBR071W	10	YHL026C	15	YOR317W	22	YOR188W	29
YDL096C	5	YDL127W	10	YIL167W	15	YPL187W	22	YPL032C	29
YDR353W	5	YDR545W	10	YIL168W	15	YAR008W	23	YPR141C	29
YEL047C	5	YEL040W	10	YJL079C	15	YBR070C	23	YPR159W	29
YER016W	5	YEL075C	10	YKR042W	15	YBR296C	23	YBL064C	30
YER152C	5	YEL076C	10	YLR041W	15	YCL023C	23	YBR053C	30
YGL027C	5	YER111C	10	YLR297W	15	YCL061C	23	YBR067C	30
YHR127W	5	YER189W	10	YML050W	15	YDL011C	23	YCL040W	30
YJL173C	5	YER190W	10	YML066C	15	YDL102W	23	YCL042W	30
YJR148W	5	YFL064C	10	YMR253C	15	YDL105W	23	YDR368W	30
YKL066W	5	YFL065C	10	YOL150C	15	YDL156W	23	YFR015C	30
YKL067W	5	YFL066C	10	YOR229W	15	YDL227C	23	YGL037C	30
YKL101W	5	YFL067W	10	YOR258W	15	YDR503C	23	YKL103C	30
YKL127W	5	YGR296W	10	YAL053W	16	YDR528W	23	YML100W	30
YKR090W	5	YHL049C	10	YBR007C	16	YER170W	23	YNL134C	30
YLR121C	5	YHL050C	10	YBR161W	16	YFL008W	23	YNL173C	30
YLR154C	5	YHR218W	10	YDL157C	16	YFR027W	23	YOR230W	30
YLR234W	5	YHR219W	10	YDL197C	16	YGL038C	23	YPR160W	30
YLR326W	5	YIL177C	10	YDL211C	16	YHR153C	23	YAR071W	31
YML012W	5	YJL225C	10	YDR144C	16	YIL025C	23	YBL023C	31
YML109W	5	YKR077W	10	YDR356W	16	YIL026C	23	YBR093C	31
YMR238W	5	YLL066C	10	YDR488C	16	YJL181W	23	YBR202W	31
YNL169C	5	YLL067C	10	YDR501W	16	YJR030C	23	YCR042C	31
YNL263C	5	YLR049C	10	YER019W	16	YJR154W	23	YDR191W	31
YOL019W	5	YLR462W	10	YER118C	16	YKL042W	23	YEL032W	31
YOR084W	5	YLR463C	10	YGL061C	16	YKL108W	23	YHR005C	31
YOR114W	5	YLR464W	10	YGL093W	16	YLR032W	23	YHR215W	31
YOR321W	5	YLR465C	10	YGL200C	16	YLR212C	23	YJL157C	31
YPR075C	5	YLR466W	10	YGR140W	16	YLR235C	23	YJL194W	31
YPR076W	5	YLR467W	10	YGR188C	16	YLR236C	23	YLR274W	31
YPR106W	5	YNL339C	10	YHR172W	16	YLR457C	23	YNL145W	31
YAL040C	6	YOL011W	10	YIL132C	16	YLR458W	23	YOR066W	31
YBR287W	6	YPL283C	10	YJL018W	16	YML102W	23	YPR019W	31
YDL039C	6	YPR202W	10	YJL019W	16	YMR048W	23	YDR055W	32
YDL138W	6	YPR203W	10	YJL091C	16	YNL225C	23	YJL159W	32
YDR001C	6	YPR204W	10	YJR006W	16	YNL273W	23	YKL163W	32
YDR077W	6	YBR088C	11	YKL165C	16	YNL289W	23	YKL164C	32
YDR157W	6	YDL003W	11	YLR343W	16	YNL304W	23	YKL185W	32
YDR337W	6	YER001W	11	YML061C	16	YOL101C	23	YNL327W	32
YFL006W	6	YER070W	11	YMR076C	16	YOR033C	23	YAR007C	33
YFL011W	6	YGR189C	11	YNL165W	16	YOR144C	23	YBL035C	33
YFR002W	6	YIL066C	11	YOR195W	16	YOR342C	23	YBR089W	33
YGL032C	6	YIL140W	11	YPL124W	16	YPR018W	23	YCL022C	33
YGL201C	6	YKR012C	11	YPL209C	16	YCR002C	24	YCL024W	33
YGR065C	6	YKR013W	11	YPL241C	16	YDR448W	24	YCL060C	33
YHR022C	6	YLR183C	11	YPL255W	16	YGL212W	24	YDR097C	33
YHR092C	6	YML027W	11	YAL022C	17	YGR124W	24	YDR309C	33
YIL072W	6	YMR199W	11	YAR018C	17	YHR006W	24	YER095W	33

ORF	Cluster #	ORF	Cluster #	ORF	Cluster #	ORF	Cluster #	ORF	Cluster #
YIL121W	6	YMR305C	11	YBR139W	17	YHR030C	24	YGR109C	33
YIL162W	6	YOL007C	11	YBR200W	17	YHR208W	24	YGR151C	33
YJL195C	6	YOL090W	11	YDL128W	17	YIL074C	24	YGR152C	33
YKL044W	6	YPL163C	11	YGL116W	17	YIL117C	24	YGR221C	33
YKL209C	6	YPL256C	11	YGR092W	17	YIL135C	24	YJL115W	33
YKR103W	6	YBL098W	12	YGR143W	17	YIL138C	24	YKL045W	33
YLR013W	6	YBL100C	12	YGR230W	17	YKL052C	24	YMR179W	33
YLR040C	6	YBR069C	12	YHR152W	17	YKL065C	24	YNL082W	33
YNL328C	6	YBR086C	12	YLR254C	17	YLR095C	24	YNL262W	33
YOL132W	6	YBR138C	12	YMR031C	17	YLR099C	24	YNL309W	33
YOR049C	6	YCL038C	12	YNL057W	17	YLR210W	24	YOL017W	33
YOR126C	6	YCL043C	12	YOL070C	17	YLR225C	24	YPL153C	33
YOR127W	6	YCR069W	12	YOL158C	17	YML117W	24	YPL267W	33
YOR270C	6	YDR276C	12	YOR313C	17	YMR055C	24	YPR120C	33
YPL095C	6	YDR432W	12	YOR314W	17	YMR278W	24	YPR175W	33
YBR008C	7	YEL065W	12	YBR083W	18	YNL003C	24		
