# Supplementary material for: Network motif-based identification of transcription factor-target gene relationships by integrating multi-source biological data
Source: BMC Bioinformatics. 2008 Apr 21;9:203. doi: 10.1186/1471-2105-9-203 (PMC2386822; doi:10.1186/1471-2105-9-203)
Supplement: Additional file 2 — Potential TFs among yeast cell cycle related genes. The data provided present TFs we identified from 800 cell cycle related genes based on their GO annotations. The genes annotated with terms related to transcription activities and DNA binding are considered as potential TFs. [file 1471-2105-9-203-S2.pdf]

Additional file 2 – Potential TFs among the yeast cell cycle related genes

We identified the genes below as potential TFs from 800 cell cycle related genes based on their GO annotations. The genes annotated with terms related to transcription activities and DNA binding are considered as potential TFs.

Gene Name	ORF	Gene Name	ORF	Gene Name	ORF
ACE2	YLR131C	KAR4	YCL055W	RFA3	YJL173C
ADA2	YDR448W	MATALPHA1	YCR040W	RFC4	YOL094C
ARP7	YPR034W	MCM2	YBL023C	RFC5	YBR087W
ASH1	YKL185W	MCM3	YEL032W	RLF2	YPR018W
CAC2	YML102W	MCM4	YPR019W	RME1	YGR044C
CBF2	YGR140W	MCM5	YLR274W	SFG1	YOR315W
CDC45	YLR103C	MCM6	YGL201C	SMC1	YFL008W
CDC6	YJL194W	MCM7	YBR202W	SPT16	YGL207W
CHA4	YLR098C	MEC3	YLR288C	STB1	YNL309W
CRP1	YHR146W	MET28	YIR017C	STB5	YHR178W
CTF4	YPR135W	MIF2	YKL089W	STP2	YHR006W
EST1	YLR233C	MIG2	YGL209W	SUT1	YGL162W
FKH1	YIL131C	MSH2	YOL090W	SWI4	YER111C
GAT3	YLR013W	MSH6	YDR097C	SWI5	YDR146C
GCR1	YPL075W	NDD1	YOR372C	TAF2	YCR042C
HCM1	YCR065W	NRM1	YNR009W	TBF1	YPL128C
HHF1	YBR009C	ORC1	YML065W	TEA1	YOR337W
HHF2	YNL030W	PHD1	YKL043W	TEC1	YBR083W
HHO1	YPL127C	PLM2	YDR501W	TEL2	YGR099W
HHT1	YBR010W	PMS1	YNL082W	TOP3	YLR234W
HHT2	YNL031C	POG1	YIL122W	TOS4	YLR183C
HMLALPHA1	YCL066W	RAD5	YLR032W	VHR1	YIL056W
HOP1	YIL072W	RAD53	YPL153C	WHI5	YOR083W
HST3	YOR025W	RAD54	YGL163C	WTM1	YOR230W
HST4	YDR191W	RAP1	YNL216W	WTM2	YOR229W
HTA1	YDR225W	RDH54	YBR073W	YHP1	YDR451C
HTA2	YBL003C	RDS2	YPL133C	YOX1	YML027W
HTB1	YDR224C	RFA1	YAR007C		
HTB2	YBL002W	RFA2	YNL312W		
