# Supplementary material for: Network motif-based identification of transcription factor-target gene relationships by integrating multi-source biological data
Source: BMC Bioinformatics. 2008 Apr 21;9:203. doi: 10.1186/1471-2105-9-203 (PMC2386822; doi:10.1186/1471-2105-9-203)
Supplement: Additional file 3 — Predicted motifs for gene clusters. The data provided present the top three enriched motifs for each gene cluster identified in this study. The motifs are predicted through promoter sequence analysis of the gene clusters using WebMOTIFS . This information helps in the validation of the NM prediction results. For example, the predicted downstream gene clusters of FKH1 all have a motif called Fork_head, GTAAACAA, in their promoter regions. This suggests that our NM inference strategy has the capability to identify the downstream target genes for TFs based on their NM assignment. The motif is annotated in Pfam database . [file 1471-2105-9-203-S3.pdf]

Additional file 3 – Predicted motifs for gene clusters

The table below presents the top three enriched motifs for each gene cluster identified in this study. The motifs are predicted through promoter sequence analysis of the gene clusters using WebMOTIFS (http://fraenkel.mit.edu/webmotifs/). This information helps in the validation of the NM prediction results. For example, the predicted downstream gene clusters of FKH1 all have a motif called Fork_head, GTAAACAA, in their promoter regions. This suggests that our NM inference strategy has the capability to identify the downstream target genes for TFs based on their NM assignment. The motif is annotated in Pfam database (http://pfam.sanger.ac.uk/).

Cluster ID	# of Predicted Motif	Predicted motifs
1	12	bZIP
	4	HSF_DNA-bind
	2	Zn_clus
2	1	E2F_TDP
	3	IRF
	1	Runt
4	6	Fork_head
	5	homeobox
	2	TBP
5	3	SRF-TF
	4	HLH
	6	Fork_head
6	1	E2F_TDP
	12	bZIP
	2	Myc_N_term
7	3	SRF-TF
	4	RHD
	2	bZIP_Maf
8	6	Fork_head
	3	myb_DNA-binding
	7	zf-C4
9	1	CBFB_NFYA

Cluster ID	# of Predicted Motif	Predicted motifs
	2	HNF-1_N
	4	RHD
Cluster ID	# of Predicted Motif	Predicted motifs
10	6	Fork_head
	4	HSF_DNA-bind
	3	PAX
11	2	bZIP_Maf
	7	zf-C4
	1	AP2-domain
12	1	E2F_TDP
	12	bZIP
	4	HSF_DNA-bind
13	1	TF_AP-2
	2	Fork_head
	4	RHD
14	4	RHD
	12	bZIP
	7	zf-C4
15	1	TF_AP-2
	4	HLH
	2	Myc_N_term
16	5	homeobox
	3	IRF
	2	bZIP_Maf
17	1	CBFB_NFYA
	4	HLH
	12	bZIP
18	3	SRF-TF
	12	bZIP
	1	TF_Otx
19	4	HLH
	2	HNF-1_N
	3	SRF-TF
20	1	E2F_TDP
	4	HSF_DNA-bind
	7	zf-C4
22	2	Myc_N_term

Cluster ID	# of Predicted Motif	Predicted motifs
	1	E2F_TDP
	4	HSF_DNA-bind
23	2	Zn_clus
	3	PAX
	1	TEA
24	12	bZIP
	4	HSF_DNA-bind
	1	E2F_TDP
25	4	HSF_DNA-bind
	3	myb_DNA-binding
	3	SRF-TF
28	3	SRF-TF
	1	AP2-domain
	2	TBP
29	1	Runt
	12	bZIP
	2	Myc_N_term
30	6	Fork_head
	1	AP2-domain
	2	Myc_N_term
31	4	RHD
	3	myb_DNA-binding
	2	MH1
32	3	SRF-TF
	5	homeobox
	1	AP2-domain
34	3	PAX
	1	Fork_head
	12	bZIP
